# Identification of key factors conquering developmental arrest of somatic cell cloned embryos by combining embryo biopsy and single-cell sequencing

**DOI:** 10.1038/celldisc.2016.10

**Published:** 2016-06-07

**Authors:** Wenqiang Liu, Xiaoyu Liu, Chenfei Wang, Yawei Gao, Rui Gao, Xiaochen Kou, Yanhong Zhao, Jingyi Li, You Wu, Wenchao Xiu, Su Wang, Jiqing Yin, Wei Liu, Tao Cai, Hong Wang, Yong Zhang, Shaorong Gao

**Affiliations:** 1 Clinical and Translational Research Center of Shanghai First Maternity and Infant Hospital, School of Life Sciences and Technology, Tongji University, Shanghai, China; 2 Graduate School of Peking Union Medical College, Beijing, China; 3 National Institute of Biological Sciences, NIBS, Beijing, China; 4 School of Life Sciences, Tsinghua University, Beijing, China

**Keywords:** embryo biopsy, Kdm4b, Kdm5b, nuclear transfer, single-cell sequencing

## Abstract

Differentiated somatic cells can be reprogrammed into totipotent embryos through somatic cell nuclear transfer. However, most cloned embryos arrest at early stages and the underlying molecular mechanism remains largely unexplored. Here, we first developed a somatic cell nuclear transfer embryo biopsy system at two- or four-cell stage, which allows us to trace the developmental fate of the biopsied embryos precisely. Then, through single-cell transcriptome sequencing of somatic cell nuclear transfer embryos with different developmental fates, we identified that inactivation of Kdm4b, a histone H3 lysine 9 trimethylation demethylase, functions as a barrier for two-cell arrest of cloned embryos. Moreover, we discovered that inactivation of another histone demethylase Kdm5b accounts for the arrest of cloned embryos at the four-cell stage through single-cell analysis. Co-injection of Kdm4b and Kdm5b can restore transcriptional profiles of somatic cell nuclear transfer embryos and greatly improve the blastocyst development (over 95%) as well as the production of cloned mice. Our study therefore provides an effective approach to identify key factors responsible for the developmental arrest of somatic cell cloned embryos.

## Introduction

Terminally differentiated somatic cells can be reprogrammed into totipotent embryos through somatic cell nuclear transfer (SCNT). Since the birth of ‘Dolly,’ the first cloned mammal [1], more than 20 mammalian species have been successfully cloned through SCNT using various somatic cell types [2, 3]. Recently, the successful reprogramming of human somatic cells by SCNT and the derivation of nuclear transfer embryonic stem cells (ntESCs) has been demonstrated to be feasible [4]. Furthermore, the derivation of human ntESCs from aged adult and patient donor cells [5, 6] holds promise for future clinical applications of therapeutic cloning. Collectively, these studies indicate that the future development prospect of SCNT is captivating.

Despite many advances that have been made, SCNT efficiency remains very low in terms of blastocyst development and the birth of full-term animals [2, 7]. In cloned embryos, abnormalities in gene expression have been observed at the two-cell stage, which corresponds to the major wave of zygotic genome activation in normal embryogenesis of the mouse [3, 8]. Moreover, SCNT embryos are usually incapable of repressing some somatic genes inherited from donor cells [3, 9, 10]. Abnormal gene reactivation can be partly rescued by the demethylation of histone H3 lysine 9 trimethylation (H3K9me3) using *Kdm4d* [8] or treatment with histone deacetylase inhibitors. As inconsistent patterns of gene misregulation have been observed in different studies, scientists have proposed that the numbers and roles of the misregulated genes determine the fate of each cloned embryo [2]; hence, identification of these decisive factors may represent a promising approach for improving cloning efficiency. The transcriptional profiles of cloned embryos at different stages have been analyzed using single-cell RNA sequencing (scRNA-seq) [11, 12]. However, SCNT embryos were not analyzed based on their developmental potency.

As a considerable percentage of cloned embryos arrest at early developmental stages [7], dissecting the molecular differences between SCNT embryos that undergo developmental arrest and those that are capable of blastocyst development may provide new insights into molecular determinants for SCNT reprogramming. To this end, we designed an efficient biopsy culture system to harvest a single blastomere from cloned embryos at the two- or four-cell stage without interrupting the developmental potency of the rest blastomere(s). Combined with scRNA-seq profiling [13], we have generated, to our knowledge, the first global transcriptome for cloned embryos with distinct development potentials. In this study, we successfully identified *Kdm4b*, an H3K9me3 demethylase, as the key factor for two-cell arrest of cloned embryos. We subsequently found that *Kdm5b*, an H3K4me3 demethylase, serves as the key factor for four-cell arrest. Co-injection of *Kdm4b* and *Kdm5b* mRNAs during SCNT restores the transcriptional profiles at two- and four-cell stage. Strikingly, these two factors significantly improved blastocyst development to over 95% as well as the success of ntESC derivation from the SCNT embryos. Our study offers an effective way to identify crucial factors responsible for SCNT embryo development, and suggests that multiple layers of epigenetic regulation impact the transcriptome resetting, and thus could have important roles in both the reprogramming and redifferentiation processes in SCNT embryos.

## Results

### Establishment of an embryo biopsy system to trace the developmental fate of SCNT embryos

**Figure 1:**
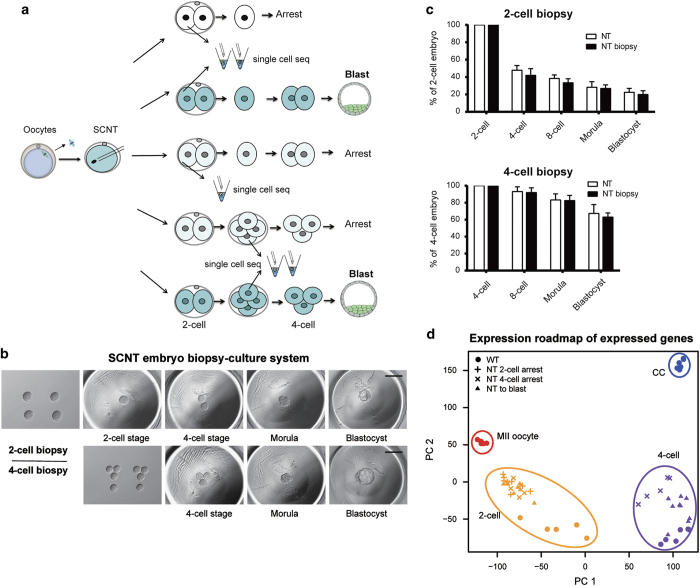
Embryo biopsy enables single-cell sequencing of the SCNT embryos with distinct developmental fates. (**a**) Schematics of blastomere biopsy and single-cell sequencing analysis for cloned embryos with different developmental fates. One blastomere of a two- or four-cell-stage cloned embryo was picked for single-cell sequencing, and the rest were cultured to trace developmental potency. (**b**) Blastomere biopsy of cleavage-stage cloned embryos and culture system for developmental potency observation. Two-cell-stage (upper row) or four-cell-stage (lower row) cloned embryos were separated by gentle pipetting. After biopsy, one or three blastomeres were placed into a depression in the aggregation plate. The remaining blastomeres could arrest or develop into blastocysts. (**c**) Preimplantation developmental potency of cloned embryos was unaffected by blastomere biopsy. The blastocyst rates of two-cell-stage (upper panel) and four-cell-stage (lower panel) embryos after blastomere biopsy were calculated and compared with untreated SCNT embryos. The rate was calculated based on the number of blastocysts at 4.5 days post activation. The data are represented as the mean±s.d. (*n*>6). (**d**) PCA showing the genome-wide expression road map in WT and SCNT embryos with distinct cell fate. Color represents different developmental stages and shape represents different types of samples. Genes with averaged FPKM⩾1 across all samples were used in the analysis.

Compared with normally fertilized embryos, a large proportion of SCNT embryos arrested at early developmental stages. To precisely dissect the molecular differences among SCNT embryos with distinct developmental potentials, we established an embryo biopsy culture system followed by scRNA-seq. In this system, we first separated live totipotent two-cell- or four-cell-stage embryos into single blastomere (Figure 1a and b). One blastomere was then harvested for scRNA-seq analysis, and the remaining blastomere(s) were further cultured to monitor the later developmental fate (see Materials and Methods for details) (Figure 1a and b).

We first confirmed that the removal of one blastomere at the two- or four-cell stage did not influence the developmental capacity of the biopsied SCNT embryos (Figure 1c). From the two-cell-embryo biopsies, we obtained three types of cloned embryos: SCNT embryos arrested at the two-cell stage (NT two-cell arrest, first row of Figure 1a), SCNT embryos arrested at the four-cell stage (NT two-cell to four-cell arrest, third row of Figure 1a) and SCNT embryos that developed into blastocysts (NT two-cell to blast, second row of Figure 1a). From the four-cell-embryo biopsies, we obtained two types of embryos: SCNT embryos arrested at the four-cell stage (NT four-cell arrest, fourth row of Figure 1a) and SCNT embryos that developed into blastocysts (NT four-cell to blast, fifth row of Figure 1a).

To obtain the molecular road map of SCNT embryos with distinct development potentials, we generated five to nine scRNA-seq profiles [13] for each classified SCNT embryo types (three types from two-cell biopsy; two types from four-cell biopsy). The *in vivo*-fertilized embryos were also harvested as wild-type (WT) control samples, and the gene expression profiles of WT embryos, MII oocytes and cumulus cells (CC) were also analyzed. We first confirmed that our RNA-seq profiles of WT embryos were consistent with previously published data [14] (Supplementary Figure S1A). The scRNA-seq profiles for blastomeres from the same type of SCNT embryos were also highly reproducible except for the NT four-cell arrest embryos, suggesting the diverse abnormality of four-cell arrest samples (Supplementary Figure S1B). Principal component analysis (PCA) indicated that gene expression profile of blastomeres from SCNT embryos was distinct from WT embryos in general (Figure 1d). Apparent unfaithful zygotic genome activation and somatic cell memory retaining can be detected in those developmental arrest SCNT embryos (Supplementary Figure S1C). This suggested that the unfaithful embryogenesis of SCNT embryos might closely correlate with the transcriptome defects in two- and four-cell stage.

### *Kdm4b* is a key factor regulating the developmental capacity of two-cell SCNT embryos

**Figure 2:**
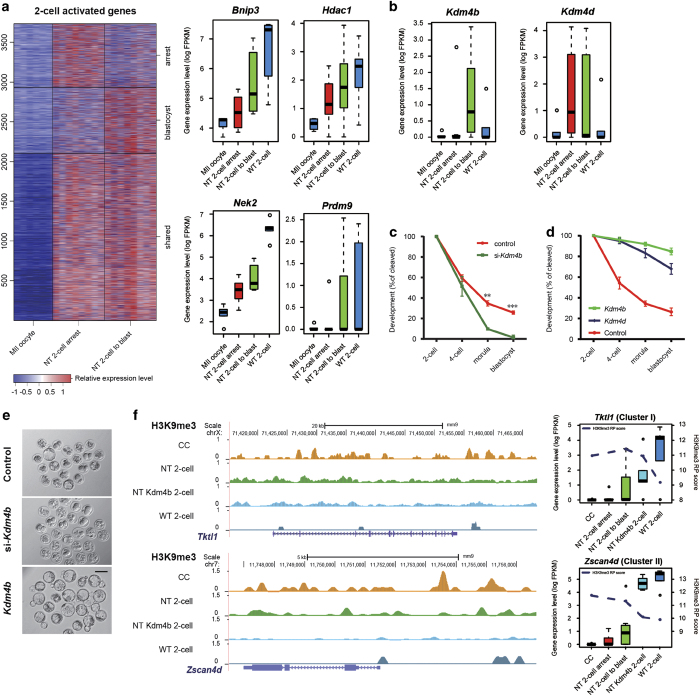
Effective identification of key factors in two-cell arrest SCNT embryos. (**a**) Impaired zygotic genome activation in SCNT two-cell-stage embryos. Heatmap showing upregulated genes for NT two-cell arrest and NT two-cell to blast relative to MII oocytes. Each row represented a Refseq transcript and was clustered using k-means, and each column represented a single-cell transcriptome and was classified by sample condition. Boxplot indicating expression level of represent chromatin modification-related genes were plotted to the right of the heatmap. (**b**) Boxplot showing the expression level of *Kdm4b* (left) and *Kdm4d* (right) in MII oocyte, WT-two-cell stage and SCNT two-cell embryos. Expression levels were quantified to FPKM with log scale. (**c**) *Kdm4b* siRNA injection dramatically reduced preimplantation development of SCNT embryos. Shown is the percentage of embryos that reached the indicated stages. (**d**) Both *Kdm4b* and *Kdm4d* mRNA injection greatly improved the preimplantation development rate of SCNT embryos. Cumulus cells were used as donor cells, and the function of *Kdm4b* was stronger than *Kdm4d*. The data are represented as the mean±s.d. (*n*>3). (**e**) Representative images of control, *Kdm4b siRNA*-injected and *Kdm4b* mRNA-injected SCNT embryos at 4.5 days post activation. (**f**) Example of *Kdm4b* target genes based on ChIP-seq data. Left, Genome browser view of the H3K9me3 signal in the *Tktl1* (Cluster I in Supplementary Figure S5B) and *Zscan4d* (Cluster II in Supplementary Figure S5B) promoter regions. Signals were smoothed by 10 pixels using the UCSC genome browser. Right, expression levels of *Tktl1* and *Zscan4d* in different types of two-cell samples. The distance-normalized H3K9me3 signal was plotted as a dashed line over the box.

To identify key candidate factors responsible for developmental arrest at the two- and four-cell stages, SCNT embryos that either arrested or proceeded to blastocyst development were compared. Considering the presence of large amount of maternal loaded transcripts at the two-cell stage, we focused on zygotic activated genes (genes upregulated in the two-cell-stage compared with MII oocytes, see Materials and Methods for details), which can separate NT two-cell arrest with NT two-cell to blastocyst by PCA (Supplementary Figure S2A). We clustered the 3 736 zygotic activated genes in NT two-cell arrest and NT two-cell to blastocyst into three groups, and focused on the group with higher expression level in NT two-cell to blastocyst samples (Figure 2a). The genes in this group, which failed to be activated in SCNT arrested embryos, may account for subsequent deficiencies in later development. We next focused on transcription factors and epigenetic regulators, as they are the major decision makers of the cell fate. We obtained 1 606 potential transcription factors and epigenetic regulators from previous publications [15, 16] and found that 79 of the blastocyst-high genes were included in the list, such as *Kdm4b, Hdac1, Prdm9* and *Msl3* (see Figure 2a and b, Supplementary Table S1), suggesting the crucial roles of epigenetic control in SCNT embryo development.

In recent studies, H3K9me3 was found to be a barrier for efficient reprogramming by SCNT. One study claimed that induction of *Kdm4b* in mouse ES cells increased *in vitro* development to cloned embryos by 30% [17], while another study overexpressed *Kdm4d*, another H3K9me3 demethylase, and improved SCNT efficiency [8]. In this study, we showed *Kdm4b*, but not *Kdm4d,* with significantly higher expression level in NT two-cell blastocyst samples than NT two-cell arrest samples (Figure 2b, Supplementary Figure S3A and B), indicating that *Kdm4b*, instead of *Kdm4d*, may function as a natural assistance for SCNT embryos to overcome the H3K9me3 barrier. We further performed gain- and loss-of-function experiments for *Kdm4b* in SCNT embryos. We first blocked the function of *Kdm4b* by injecting short interfering RNAs (siRNAs) targeting *Kdm4b* into enucleated MII oocytes (Supplementary Figure S4A), and found that blastocyst development of SCNT embryos was greatly reduced when *Kdm4b* was repressed (Figure 2c and e). In contrast, the efficiency of SCNT was greatly improved by injecting *Kdm4b* mRNA into enucleated MII oocytes prior to SCNT, and the efficiency is even higher than those injected with *Kdm4d* mRNA (Figure 2d and e, Supplementary Figure S4B). Overall, these results indicated that *Kdm4b* could function as a natural key regulator during SCNT embryo development.

To confirm the underlying molecular mechanism of *Kdm4b* regulation, by immunostaining, we found that the overexpression of *Kdm4b* indeed promoted the removal of H3K9me3 in one- and two-cell-stage SCNT embryos (Supplementary Figure S5A). We then applied ultra-low-input native ChIP-seq [18] to determine the genome distribution of H3K9me3 in CC and two-cell-stage embryos (WT, SCNT, SCNT injected with *Kdm4b*) with 500 to 1000 cells. We found that 7 248 genes (Cluster I and Cluster II in Supplementary Figure S5B and C) showed resisted CC-liked H3K9me3 signal at promoters in two-cell stage SCNT embryos, and the resisted H3K9m3 signal reduced in *Kdm4b*-injected SCNT two-cell compared with that in CC and SCNT two-cell embryos (examples shown in Figure 2f). We then performed scRNA-seq in two-cell-stage blastomeres from *Kdm4b*-injected SCNT embryos (NT *Kdm4b* two-cell) to check the corresponding gene expression level change. As expected, genes in Cluster I and Cluster II were largely reactivated in NT *Kdm4b* two-cell samples (Figure 2f and Supplementary Figure S5D). This finding supported our hypothesis that H3K9me3 in SCNT embryos genome functions as a major barrier for cloned embryos and it cannot be removed appropriately in arrested two-cell stage SCNT embryos. Furthermore, we demonstrate that *Kdm4b* functions as a natural H3K9me3 barrier eraser during SCNT embryo development to facilitate the zygotic genome activation.

### *Kdm5b* is a critical factor in the four-cell arrest of SCNT embryos

**Figure 3:**
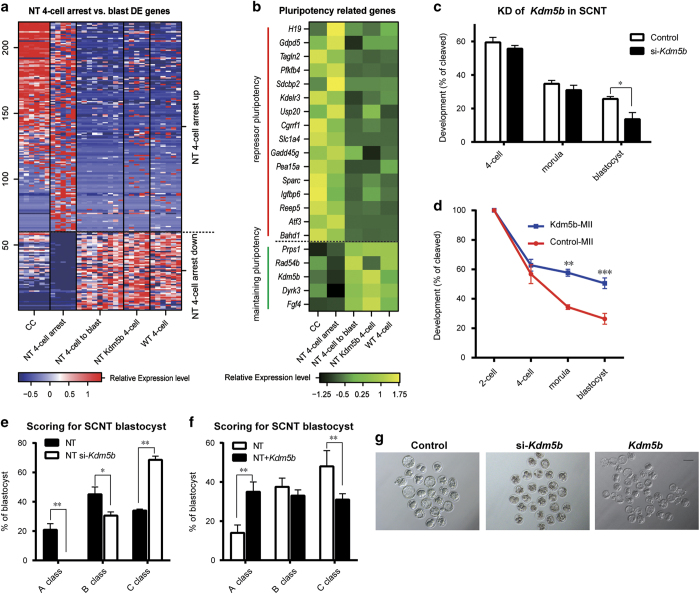
*Kdm5b* is a crucial factor identified in the four-cell arrest SCNT embryos. (**a**) Heatmap of 219 differentially expressed genes between NT four-cell-arrested and NT four-cell blast samples (see Materials and Methods). The relative expression levels of CC, NT four-cell arrest, NT four-cell to blast, NT *Kmd5b* four-cell and WT four-cell samples are plotted. Upregulated (59) and downregulated (160) genes were plotted separately. Each row represented a Refseq transcript and each column represented a single-cell sample. Expression level of each transcript was normalized based on row *Z*-score. (**b**) Expression levels of 21 pluripotency genes from the differential expressed genes in **a**. Each row represented a Refseq transcript and each column represented the mean of the expression level of the same sample type, normalized based on row *Z*-score. (**c**) Knockdown of *Kdm5b* slightly reduced the preimplantation development of SCNT embryos. Shown is the percentage of embryos that reached the indicated stages. (**d**) Injection of *Kdm5b* improved the rate of four-cell to blastocyst development. (**e**) Knockdown of *Kdm5b* in SCNT embryos inhibited the formation of expanded blastocysts. Control and *Kdm5b* siRNA-injected SCNT blastocysts at 4.5 days post activation were graded into three groups depending on their size and blastocoelic hatching. *P<0.05, **P<0.01, ***P<0.001 according to Student’s *t*-test. (**f**) Injection of *Kdm5b* mRNA promoted the formation of expanded blastocysts, similar to **e**. (**g**) Representative images of control, *Kdm5b siRNA*-injected and *Kdm5b* mRNA-injected SCNT embryos at 4.5 days post activation.

In addition to two-cell arrest, four-cell arrest is also frequently observed in SCNT embryos. We next sought to investigate the transcriptome defects that may account for the developmental arrest at four-cell stage. PCA using all genes showed clear separation between NT four-cell arrest samples and NT four-cell to blast samples, indicating the remarkable discrepancy of arrest SCNT embryos (Supplementary Figure S2B). We identified 219 differential expressed genes between those two groups of SCNT embryos, of which 159 genes were highly expressed in four-cell-arrest samples, and 60 genes were not properly reactivated (Figure 3a).

As previous studies indicated, expression of pluripotency-related epigenetic modifiers can enhance blastocyst formation [19], while somatic genes, especially repressors of pluripotency, can impede developmental potency. To identify the key candidate factors in the four-cell stage for blastocyst development of SCNT embryos, we compared the above differential expressed genes list to the pluripotency-related genes (maintaining pluripotency and repressors of pluripotency, Figure 3b) identified through RNA-seq of ESC-derivation tracing [20]. Subsequently, 16 genes in the cluster of genes highly expressed in the four-cell-arrest samples were identified as repressors of pluripotency, whereas 5 genes in the cluster of genes not activated in the four-cell-arrest embryos were identified as critical factors for maintaining pluripotency (Figure 3b). Among these genes, we found that another epigenetic modifier, *Kdm5b* (Supplementary Figure S3C), failed to be activated in the four-cell-arrested SCNT embryos (Figure 3b and Supplementary Figure S4B).

To determine the role of *Kdm5b* in the development of SCNT embryos, we injected *Kdm5b* siRNA into enucleated MII oocytes and found that the developmental efficiency of SCNT embryos was reduced, while the rate of high-quality blastocyst development was largely reduced (Figure 3c, e and g, Supplementary Figure S4A). Moreover, when *Kdm5b* was overexpressed in SCNT embryos, although the rate of two-cell arrest remained high, the rate of four-cell arrest was significantly reduced. The efficiency of blastocyst formation was increased, and more importantly, the quality of the blastocysts was greatly improved by *Kdm5b* overexpression (Figure 3d, f and g, Supplementary Figure S4B). Consistent with the improvement of SCNT efficiency, scRNA-seq of *Kdm5b*-rescued four-cell-stage samples showed restored expression level compared with NT four-cell arrested samples (Figure 3a and b). These data suggest that *Kdm5b* is a novel important epigenetic factor whose proper activation can rescue four-cell arrest and improve blastocyst quality in SCNT embryos.

### The combination of *Kdm4b* and *Kdm5b* improves SCNT blastocyst development to over 95%

On the basis of the above results, our approach identified previously reported key factor *Kdm4b* and novel factor *Kdm5b* responsible for two-cell and four-cell stage arrests during SCNT embryo development separately. We next investigated whether the combination of these two factors could further improve SCNT embryonic development. First, we found that the concentration of *Kdm4b* mRNA injected greatly affected the developmental potential of SCNT embryos. The higher concentration of *Kdm4b* (200–800 ng μl^−1^) injected showed detrimental effect on the implantation and birth rate of SCNT mice, while lower concentration (<50 ng μl^−1^) showed less effect on the improvement of *in vitro* developmental potential of SCNT embryos (Supplementary Figure S6A-C). Therefore, the injection concentration of *Kdm4b* in our experiment was optimized to 100 ng μl^−1^.

**Figure 4:**
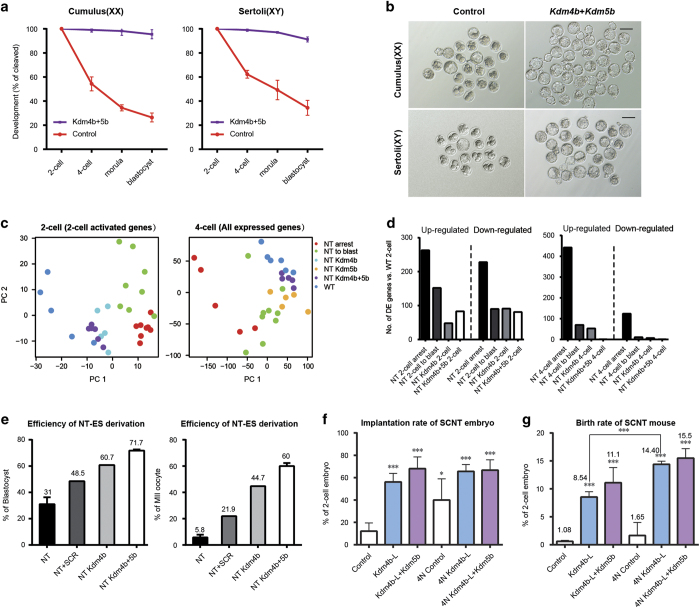
*Kdm4b* and *Kdm5b* co-injection greatly facilitates the development of SCNT embryos. (**a**) Co-injection of *Kdm4b+5b* greatly improved the preimplantation development rate of SCNT embryos. Both cumulus cells (left panel) and Sertoli cells (right panel) were used as donor cells. (**b**) Representative images of SCNT embryos injected with *Kdm4b+5b* mRNA. (**c**) PCA on SCNT embryos with single mRNA rescued, *Kdm4b+5b* co-injected and WT embryos at two-cell and four-cell stage. Zygotic genome activation genes were used in two-cell analysis and all expressed genes were used in four-cell-stage analysis. Co-injected SCNT embryos mostly resembled the WT embryos in each stage. (**d**) The number of differentially expressed genes was reduced by *Kdm4b+5b* mRNA injection in NT two-cell samples (left panel) and four-cell samples (right panel). (**e**) Injection of Kdm4b and Kdm4b+5b improved the efficiency of ntESC derivation. The efficiency was calculated on the basis of the total number of blastocysts (left panel) in ntESC derivation or receipt MII oocytes (right panel) in SCNT. (**f**) Injection of *Kdm4b* alone or *Kdm4b+5b* co-injection greatly improved the implantation rates of SCNT embryos and NT-tetraploid-aggregated embryos. The data are represented as the mean±s.d. (*n*> 3). **P<0.01, ***P<0.001 according to Student’s *t*-test or the Holm-Sidak test (for analysis of variance). (**g**) Injection of *Kdm4b* alone or *Kdm4b+5b* co-injection greatly improved the birth rates of SCNT embryos and NT-tetraploid-aggregated embryos.

We then injected a mixture of *Kdm4b* and *Kdm5b* mRNAs into recipient MII oocytes before SCNT to examine their combination effects on the gene expression profile and developmental potential of cloned embryos. Surprisingly, almost all SCNT embryos developed to the blastocyst stage (>95%); similar results were observed when sertoli cells were used as donor cells (Figure 4a and b). Kdm4b and Kdm5b co-injection also restored the gene expression profile at two-cell and four-cell stages. PCA on scRNA-seq samples of *Kdm4b* and *Kdm5b* co-injected embryos revealed that their expression profile was shifted toward a WT-like state compared with the NT arrest or to blastocyst samples, or the samples injected with *Kdm4b* or *Kdm5b* individually (Figure 4c). The number of differentially expressed genes was reduced by *Kdm4b+5b* mRNA injection in NT embryos (Figure 4d). These results suggest that these two histone demethylases exert a synergistic effect on SCNT reprogramming and that their function might be conserved in SCNT performed with different donor cells.

Therapeutic cloning requires the derivation of patient-specific ESCs from SCNT blastocysts. Therefore, we next examined the efficiency of ntESC derivation using the SCNT blastocysts produced with *Kdm4b* and *Kdm5b* overexpression. Treatment with Scriptaid (SCR), which was an histone deacetylase inhibitor and has been widely used to improve cloning efficiency and ntESC derivation in multiple species [21], was used as positive control. We first performed SCNT with the following four types of treatments: injection of GFP as a control, injection of *Kdm4b* mRNA, co-injection of *Kdm4b* and *Kdm5b*, and treatment with SCR. The ntESCs were subsequently derived following standard protocols and further passaged (Supplementary Figure S6D). Compared with blastocysts cultured on feeder cells, the efficiency of ntESC derivation increased from 31 to 48.5% with SCR treatment. Strikingly, ntESC derivation was further improved by *Kdm4b* injection (60%) and *Kdm4b*+*Kdm5b* co-injection (71%) (Figure 4e). Most importantly, the efficiency was greatly improved by *Kdm4b*+*Kdm5b* co-injection when calculations were based on the total number of MII oocytes used for SCNT (5.8 vs 60%) (Figure 4e).

As the major deficiency in SCNT is attributable to impaired trophectoderm (TE) [22], tetraploid (4N) embryo complementation can be used to improve the birth rate of SCNT mice. We aggregated 4N embryos with control, *Kdm4b*-injected or *Kdm4b*-*Kdm5b*-co-injected SCNT embryos and transferred the chimeric embryos to pseudo-pregnant mothers at the blastocyst stage. Consistent with previous studies, 4N-complemented control embryos demonstrated higher implantation rates than directly transferred control SCNT embryos, which suggests that the TE in control samples does exhibit serious deficiencies with respect to implantation (Figure 4f). However, when we used the *Kdm4b*-injected or *Kdm4b*-*Kdm5b*-co-injected SCNT embryos, the implantation rates were much higher than the directly transferred control SCNT embryos, but comparable with the 4N-complemented embryos (including control, *Kdm4b*-injected or *Kdm4b-Kdm5b*-co-injected) (Figure 4f). These results suggested that the implantation ability of *Kdm4b*-injected and *Kdm4b*-*Kdm5b*-co-injected SCNT TE was greatly improved relative to control SCNT TE.

We further evaluated the generation of full-term animals using the SCNT embryos produced with different treatments. We found that the combination of *Kdm4b* with *Kdm5b* could lead to over 11% of cloned embryos developing into live animals (Figure 4g and Supplementary Figure S6E). Similarly, high developmental efficiency of 4N-complemented embryos also revealed the positive effects of *Kdm4b* and *Kdm5b* on the SCNT embryos (Figure 4g and Supplementary Figure S6F). Taken all together, the combination of optimized *Kdm4b* with *Kdm5b* could greatly improve the full-term development of SCNT embryos.

### Single-cell RRBS-seq reveals DNA methylation abnormality rescue upon *Kdm4b* and *Kdm5b* injection

**Figure 5:**
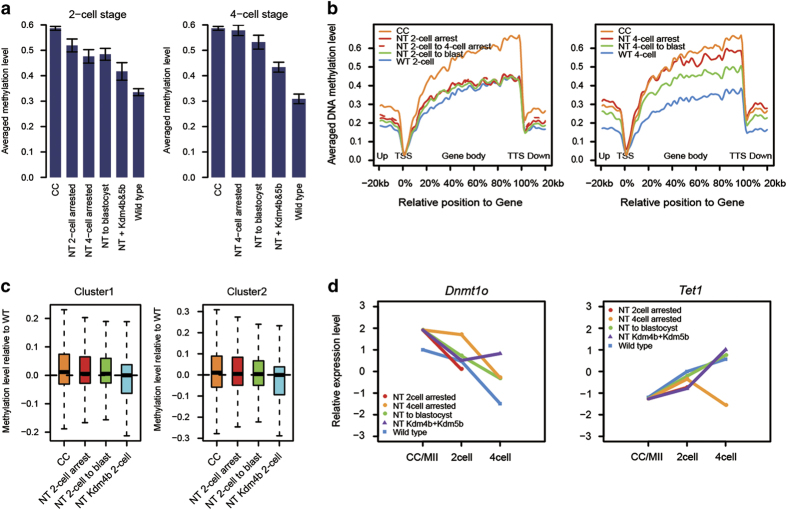
Combination of embryo biopsy and single-cell RRBS-Seq reveals abnormal DNA methylation in preimplantation SCNT embryos. (**a**) Global DNA methylation levels of SCNT samples were higher than those of WT. The average DNA methylation levels of the indicated SCNT and WT samples were calculated and compared. Each bar represents the mean of global averaged methylation level of RRBS samples from same sample type, error bar stands for the standard derivation of global averaged methylation level for each sample. (**b**) Average DNA methylation levels were determined along the gene bodies, 20 kb upstream of the transcription start sites (TSS) and 20 kb downstream of the transcription terminal sites (TTS) for all RefSeq genes. SCNT two-cell stage samples (left panel) and four-cell stage samples (right panel) were plotted separately. Methylation levels were calculated based on 1kb bins (up/downstream of TSS/TTS) or percentile binned gene body region, see Materials and Methods for detailed calculation. (**c**) Boxplot showing promoter methylation levels relative to WT two-cell samples of Cluster I and Cluster II transcripts in Supplementary Figure S5B. Promoter methylation levels were calculated based on the averaged methylation level of CpG sites located within ±2 kb around TSS. Methylation level for the SCNT samples were subtracted by the methylation level of WT two-cell for visualization. (**d**) Relative expression level of *Dnmt1* and *Tet1* genes for SCNT and WT samples. Expression levels were quantified using FPKM and then normalized based on Z-score of different sample types. Transcript with overall highest FPKM was used as the FPKM of the gene.

High levels of DNA methylation in cloned embryos have been observed across species [3, 23, 24], and aberrant DNA methylation patterns could be detected even after implantation [7]. Facilitated by our embryo biopsy system along with recently developed single-cell Reduced Representation Bisulfite Sequencing (RRBS)-Seq [25], we could generate methylome profiles of two- and four-cell-stage embryos with distinct fate to better elucidate the underlying mechanism (Supplementary Figure S7A and B). Donor CCs and WT embryo samples were also analyzed as controls. We confirmed that at two- and four-cell stage, the SCNT samples possessed generally higher methylation level than the corresponding WT embryos especially in arrested samples (Figure 5a). The averaged methylation levels on gene body regions of four-cell-stage SCNT embryos were significantly increased, particularly in the SCNT four-cell-arrested samples, which almost resembled the trend of donor CC cells (Figure 5b). The negative correlation between DNA methylation and gene expression suggests that aberrant expression profile in arrested SCNT embryos might be partly caused by high DNA methylation on promoters (Supplementary Figure S7C). The precise mechanism deserves to be further investigated in the future.

We next sought to investigate whether *Kdm4b* and *Kdm5b* co-injection could reverse the abnormal methylation in SCNT embryos. The averaged methylation level of SCNT embryos was partially rescued by *Kdm4b* and *Kdm5b* co-injection (Figure 5a), but still higher than WT embryos at the same stage. Comprehensive analysis was performed to explore the potential correlation between H3K9me3, DNA methylation and transcription. Consistent with the studies in other system, inverse correlation was found between H3K9me3 on promoter and gene expression in CC and two-cell-stage embryos, and positive correlation was found between H3K9me3 and DNA methylation on promoter (Supplementary Figure S8A and B). Moreover, the genes with rescued H3K9me3 by Kdm4b injection showed reduced DNA methylation compared with non-injected SCNT two-cell-stage embryos (including NT two-cell arrest and NT two-cell to blast, Figure 5c).

## Discussion

SCNT-mediated reprogramming can produce blastocysts as well as full-term individuals, with clear promise for therapeutic applications and animal cloning. Although SCNT technology has advanced in the last decade, developmental efficiency remains extremely low, and the molecular mechanism underlying SCNT embryo arrest remains largely undefined [3, 21]. Single-cell sequencing enables the analysis of genome-wide information from rare samples; therefore, this technique could be particularly helpful for identifying molecular mechanisms involved in the success of SCNT.

In the present study, we established an efficient SCNT embryo biopsy culture system to obtain two- and four-cell-stage single blastomeres from SCNT embryos with ascertainable developmental fates. Taking advantage of single-cell transcriptome sequencing, we were able to generate the transcriptome transition road map of SCNT embryos and precisely dissect the molecular differences that may be responsible for the developmental arrest of cloned embryos. A comprehensive analysis at the two-cell stage embryos identified a key factor, *Kdm4b*, which failed to express in two-cell-arrested embryos. Although a similar effect of *Kdm4d* was recently reported [8], previous studies were mostly based on the observations of failing to remove H3K9me3 in reprograming-resistant regions, which is a rather indirect and inefficient way in uncovering key regulators.

We further identified *Kdm5b*, a demethylase for H3K4me3, as an inducer of the four-cell-to-blastocyst switch. Injection of *Kdm5b* did not affect two-cell arrest but did rescue four-cell arrest and facilitated the formation of high-quality blastocysts. Our finding, for the first time, proved that not only the repressive epigenetic marks, but also active epigenetic markers had to be removed through SCNT. Co-injection of *Kdm4b* and *Kdm5b* remarkably improved the developmental rate as well as the efficiency of ntESC derivation than individual rescue, suggesting that different epigenetic modifications may interact with each other to maintain the proper development of embryo development.

In addition to the RNA transcriptome road map, we also successfully combine the embryo biopsy system with single-cell RRBS to generate DNA methylomes for SCNT embryos with distinct developmental fates. We found abnormal high levels of DNA methylation in two- and four-cell-stage SCNT embryos. In opposition to the demethylation process during the normal embryo development [6, 26, 27], the SCNT four-cell samples are relatively hyper-methylated compared with two-cell embryos. The expression of *Dnmt* and *Tet* family enzymes suggests that the different expression level of *Dnmt1* and *Tet1* may have roles in the aberrant DNA methylation in arrested SCNT embryos (Figure 5d, Supplementary Figure S8C and D). *Kdm4b* and *Kdm5b* co-injection could successfully reduce the abnormal methylation level in SCNT embryos, suggesting the interplay between histone modification and DNA methylation. It should be noted that, the DNA methylation road map also allows us to discover the key regulators from a different view. Future work is needed to validate and emphasize the roles of candidate genes in SNCT embryo development.

The low efficiency of SCNT has been widely observed and might be conserved in all mammals. It will be critical to test whether our approach could also improve the efficiency of SCNT in other species, especially in human. Overall, our study provides important clues for the further exploration of additional key factors that regulate SCNT-mediated reprogramming and may ultimately be used in therapeutic cloning.

## Materials and Methods

### Mice and donor cells

B6D2F1 (C57BL/6×DBA2) mice were used as oocyte donors. ICR adult females were used for the embryo transfer recipients. All mice were housed in the animal facility of Tongji University. B6D2F1 female mice (8–10 weeks old) were super-ovulated by the sequential injection with 7 IU each of pregnant mare serum gonadotropin and human chorionic gonadotropin (San-Sheng pharmaceutical Co. Ltd., Ningbo, China). Cumulus-oocyte complexes were collected at 14 h after human chorionic gonadotropin injection and treated with bovine testicular hyaluronidase (Sigma, St Louis, MO, USA) to obtain CC. Sertoli cells were collected from testes of 4–5-day-old BDF1 male mice as previously described [28]. Briefly, testicular masses were incubated in 0.1 mg ml^−1^ collagenase (Sigma) and gently suck with a straw for 10 min at 37 °C followed by 5 min treatment with 0.05% Trypsin (Sigma). After adding 10 % fetal bovine serum-phosphate-buffered saline (PBS) to terminate digestion, the dissociated sertoli cells were suspended in HCZB medium for use.

### Nuclear transfer

MII oocytes were retrieved at 14 h after human chorionic gonadotropin injection. The oocytes were enucleated in a chamber containing oil-covered HCZB supplemented with 5 μg ml^−1^ cytochalasin B (Sigma) by a blunt Piezo-driven pipette (PrimeTech, Osaka, Japan) on a heating stage (37 °C) of an Olympus inverted microscope (Tokyo, Japan). The donor cells were transferred into enucleated oocytes by direct injection, and activated by 5 h incubation in 1 mM SrCl_2_ in Ca^2+^-free CZB and 5 μg ml^−1^ cytochalasin B as described [29]. Reconstructed embryos were thoroughly washed and cultured in G1 and G2 medium (Vitrolife, Göteborg, Sweden) with amino acids at 37 °C under 5% CO_2_ in air. In the SCR-treated SCNT experiment, SCR was added to the culture medium since the beginning of the activation for a total of 10 h with the working concentration of 5 nM.

### *In vitro* transcription of mRNA and mRNA injection

WT cDNAs for Kdm4a, Kdm4b, Kdm4d, Kdm5b and GFP were cloned into T7-driven vectors and synthesized with the mMESSAGE mMACHINE T7 Ultra Kit (Life Technologies, Grand Island, NY, USA) according to the manufacturer’s instructions. The storage concentration of each mRNA was optimized to 800 ng μl^−1^. The integrity of manufactured mRNA was confirmed by electrophoresis with formaldehyde gels. Enucleated oocytes or 5 h-post-activation SCNT embryos were injected with approximately 10 pl of mRNA using a Piezo-driven micromanipulator. GFP mRNA was used as a control. In some experiments, the mRNA was diluted before injection.

### Knockdown of Kdm4b and Kdm5b in cloned embryos

SiRNAs against mouse *Kdm4b* and *Kdm5b* were diluted in nuclease-free water to a final concentration of 5 μM. Enucleated oocytes were injected with approximately 10 pl of 5 μM siRNAs for *Kdm4b* or *Kdm5b* using a Piezo-driven micromanipulator. After incubation for 30 min in CZB, the donor cells were transferred into the enucleated oocytes by direct injection.

### Derivation of ntES cells

For ntESCs derivation, the zona pellucida of the cloned blastocysts was removed by 0.5% pronase E (Sigma). Embryo was seeded onto feeder cells in a 96-well plate. The ES cells derivation medium contains Knockout Dulbecco's modified Eagle's medium supplemented with 20% Knockout Serum Replacement (Gibco, Waltham, MA, USA), 1 mM L-glutamine (Merk, Millipore, Billerica, MA, USA), 0.1 mM mercaptoethanol (Merk, Millipore), 1% nonessential amino acid stock (Merk, Millipore), nucleosides (100×, Merk, Millipore), 1500 U ml^−1^ LIF (Merk, Millipore), 3 M CHIR99021 (Merk, Millipore) and 1 M PD0325901 (Merk, Millipore). Colonies formed with culturing for 7–10 days, and were picked and transferred for cell passage. The expansion of ES cells was performed by routine culture.

### Embryo transfer

The two-cell stage control or injected SCNT embryos were transferred into the oviduct of pseudo-pregnant female mice. Cesarean section was carried out at day 19.5 and the surviving pups were fostered by lactating ICR females.

### Aggregation of cloned embryo with 4N embryos

The 4N embryos were first produced by the electrofusion of two-cell-stage embryos that were collected from ICR mice. One zona-free four-cell-stage SCNT embryo was aggregated with two zona-free four-cell-stage 4N embryos in depression wells. The aggregated embryos were cultured for 28–32 h to reach the blastocyst stage. The aggregated embryos were then transferred into the oviduct of pseudo-pregnant female mice. Cesarean section was carried out at day 19.5, and the pups were fostered by lactating ICR mothers.

### Immunofluorescent staining

Reconstructed embryos were fixed with 4% paraformaldehyde (Sigma) for 15 min and then permeabilized with 0.2% Triton X-100 for 15 min at room temperature. The samples were blocked with 1% bovine serum albumin (BSA) (Sigma) at 37 °C for 1 h and then incubated with the primary antibodies against Nanog (1:500, Santa Cruz, Dallas, TX, USA), Cdx2 (1:500, Abcam) or H3K9me3 (1:500, Abcam, Cambridge, UK) overnight at 4 °C. After washing three times with PBS, the samples were incubated with the appropriate secondary antibodies at 37 °C for 1 h. The nuclei were stained with 4',6-diamidino-2-phenylindole (Millipore). All stained samples were observed using a LSM 510 META microscope (Zeiss, North York, ON, Canada).

### Reverse transcription and quantitative RT-PCR analysis

To analyze the knockdown efficiency of siRNA, total RNA of 20 four-cell stage embryos were purified using RNeasy mini kit (QIAGEN, Dusseldorf, Germany, 74104) according to the manufacturer’s instruction. The cDNA was synthesized by a reverse transcription system (Promega, Madison, WI, USA). Quantitative RT-PCR was performed using a SYBR Premix Ex Taq (Takara, Kusatsu, Japan) and signals were detected with ABI7500 Real-Time PCR System (Applied BioSystems, Marsiling, Singapore). Gapdh was used as an endogenous control. Primers are shown in Supplementary Table S2.

### Blastocyst grading

The SCNT blastocysts were graded at E4.5 and the grading of three classes was mainly based on the size and blastocoelic hatching ability. A: hatched blastocyst, B: fully expanded blastocysts, C: not fully expanded blastocysts.

### Biopsy culture system and sample harvest for single-cell sequencing

In the biopsy culture system, the zona pellucida of WT, SCNT or mRNA-injected SCNT embryos was removed with 0.5% pronase E (Sigma). For two- or four-cell stage embryos, one blastomere was removed by gentle pipetting using a fire-polished glass needle with an inner diameter of 120 μm after incubation in Ca^2+^-free CZB. The separated blastomeres were transferred to harvest for single-cell analysis, and the rest were cultured in an aggregation plate with 5% CO_2_ at 37 °C until the blastocyst stage. The tight junctions of TE cells and ICM cells were separated by gentle pipetting in a pipette with a diameter of 40–60 μm after removal of the zona pellucida and incubation in Ca^2+^-free CZB. All samples were washed three times in 0.5% BSA-PBS (Sigma) solution before they were placed into PCR tubes.

### Single-cell RNA-Seq library generation

The single-cell RNA-seq method followed previously published studies [13, 30]. In brief, the harvested single blastomeres, ICM or TE, were washed several times in 0.5% BSA-PBS (Sigma) solution and subsequently picked and transferred into lysate buffer by a mouth pipette. Diluted ERCC mix (Life Technologies 4456740) was added in lysis buffer as spick-in for each sample. Reverse transcription was performed directly on the cytoplasmic lysate. Terminal deoxynucleotidyl transferase was then used to add a poly(A) tail to the 3′ end of the first-strand cDNAs. The total cDNA library of the single cell was then amplified by 18–20 cycles for the library construction.

The amplified cDNA was fragmented using Covaris sonicator (Covaris S220, Woburn, MA, USA). To generate the sequence libraries, the TruSeq Library Prep Pooling kit (Illumina 15042173, San Diego, CA, USA) or NEBNext Ultra DNA Library Prep Kit (New England Biolabs E7370, Ipswich, MA, USA) was used following the manufacturer’s instructions. All adapters with index-barcode in this project also including the RRBS and ULI-NChIP-seq were diluted (or not) from the adapters offered by TruSeq Library Prep Pooling kit. Paired-end 100-bp or 125-bp sequencing was further performed on a HiSeq 2500 or 2000 (Illumina) at the National Institute of Biological Sciences, Peking University and Berry Genomics Corporation.

### Single-cell RRBS library generation

Samples for single-cell RNA-seq were also used in single-cell RRBS analysis. The preparation of the RRBS library followed a previously published study [25]. In brief, the DNA from the nuclei of the indicated samples were extracted, with digestion of nuclear proteins. The DNA was further treated with *Msp*I digestion (Fermentas, Waltham, MA, USA), end repair, dA tailing, adaptor ligation, and bisulfite conversion in a one-tube reaction. The MethylCode Bisulfite Conversion Kit (Invitrogen, Carlsbad, CA, USA, MECOV-50) was used for bisulfite conversion and purification. Unmethylated lambda DNA (Fermentas) was added to the gDNA sample before *Msp*I digestion to monitor the bisulfite conversion rate. The converted libraries were further purified with Agencourt AMPure XP beads (Beckman A63881, Brea, CA, USA) and amplified by two-round PCR enrichment. After amplification, size-selection was performed on the 12% native polyacrylamide TBE gel to obtain the 200–500 bp DNA fragments. Paired-end 100-bp or 125-bp sequencing were further performed on HiSeq 2500 or 2000 (Illumina) at the Peking University and Berry Genomics Corporation.

### ULI-NChIP-seq

For ULI-NChIP-seq, 500–1000 cells were used per immunoprecipitation reaction. SCNT or WT embryos in two-cell stage were treated with 0.5% Pronase E (Sigma) to remove the zona pellucida. And the polar bodies were removed by gently pipetting in a pipette after incubation with Ca^2+^-free CZB. CCs were collected following the protocol of donor-cell preparation for SCNT. All isolated cells were washed three times in 0.5% BSA-PBS (Sigma) solution to avoid any possible contaminant. The procedure of ULI-NChIP was carried out as previously described [18]. And 1.5 μg of histone H3K9me3 antibody (Active Motif, Carlsbad, CA, USA, 39161) were used for each immunoprecipitation reaction.

The sequence libraries were generated using the TruSeq Library Prep Pooling kit (Illumina 15042173), following the manufacturer’s instructions. Paired-end 100-bp sequencing was further performed on HiSeq 2500 or 2000 (Illumina) at the National Institute of Biological Sciences and Peking University.

### Sequencing read trimming and alignment

All of the RNA-Seq sequencing reads were first mapped to mm9 reference genome using Tophat (v 1.3.3) with default parameters [31], and then evaluated using RseQC(v 2.3.4) to remove low quality samples [32], samples with mapping efficiency less than 30% or mapped reads count less than 2 M, and intron reads percentage greater than 10% were discarded for downstream analysis. Gene expression for each sample was quantified to FPKM (fragments per kilobase of exon model per million mapped reads) using Cufflinks (v 1.2.0) to eliminate the effects of sequencing depth and transcript length [33]. For bisulfite sequencing, all the pair-end RRBS samples were first processed using TrimGalore(v 0.3.3) with parameter ‘--phred33 --fastqc --paired –rrbs’ to trim the low quality reads, adaptors and end-repair effect, and then all the trimmed reads were mapped to a combined genome with mm9 and 48052 lambda sequence, using bsmap(v 2.89) with parameter ‘-D C-CGG -w 100 -s 12 -v 0.1 -m 32 -× 1000 –R’ [34]. Methylation level of each CpG site was estimated using mcall(v 1.3.0) with default parameters [35], CpG sites with read depths ⩾1 were counted as total CpG coverage of the sample, bilsulfite conversion ratio for each sample was calculated using unmethyalted CpGs divided by total CpGs detected in lambda genome. All ChIP-Seq samples were mapped to mm9 reference genome using bwa (v 0.7.12) mem command [36]; reads that have map quality score less than 30 were removed from downstream analysis. Data quality was shown in Supplementary Table S3.

### ERCC spike-in normalization

All of the RNA-Seq samples were mapped to ERCC synthetic spike-in genome (http://tools.invitrogen.com/downloads/ERCC92.fa) and quantified to FPKM for each spike-in RNA using cufflinks. FPKM of spike-in RNAs were first fit into linear regression model with ERCC mix concentration to evaluate the sample quality; almost all the samples have R square greater than 0.95 (data not shown), indicating accurate detection of amplified spike-in RNAs. We then perform linear regression on FPKM of spike-in RNAs between two different samples, and the co-efficients were used to normalize FPKM of Refseq genes [37]. All samples were normalized to the first replicate of NT two-cell arrest samples for downstream analysis.

### Transcriptome clustering and differential expression analysis

PCA for RNA-Seq samples were implemented using R function prcomp, only genes with averaged FPKM ⩾1 were used in the analysis. Differential expression analysis were conducted by edgeR (v 3.10.2) using ERCC normalized read counts [38]. For each comparison, genes with a Benjamini and Hochberg-adjusted *P* value (false discovery rate) <0.05 and a mean fold change of >1.5 were called differentially expressed. K-means clustering were performed on differentially expression genes to classify expression groups and heatmaps were generated for visualization. All analyses were performed using customized R script.

### Genomic annotations and methylation level calculation

Mouse promoters were defined as ±2 kb around the transcription start sites (TSS), and were separated into high CpG density promoters, intermediate CpG density promoters and low CpG density promoters based on the CpG density and GC content as previously defined [39]. Annotations of LINE, LTR and SINE elements were downloaded from the UCSC genome browser (mm9) Repeat Master tracks.

Global methylation level of each RRBS data set were calculated on the basis of the averaged methylation level of covered CpGs; only CpGs covered by ⩾3 reads were used in the analysis. Although the coverage of each RRBS data set varied between samples (10~35% of the total RRBS sites for single-cell RRBS samples, 37~86% for pooled RRBS samples), the variance of methylation level within the same sample type was not significant and not affected by the different coverage.

To analyze the methylation profile around genes, mm9 refseq genes were split to 100 bins with equal proportion, the upstream and downstream 20 Kb of the genes were split into 20 bins with the length of 1 Kb. Only CpGs covered by ⩾3 reads were used in the analysis. For each annotated genomic regions, the DNA methylation level was calculated as the average DNA methylation level of all CpG sites located in the region.

### ChIP-Seq peak calling and signal normalization

H3K9me3 ChIP-Seq peaks were called using MACS2(v 2.0.10.20131216) with the parameters (macs2 callpeak –n Sample -g mm -B -q 0.05 --nomodel --broad --shiftsize=73 --SPMR) relative to input samples [40]. ChIP-seq signal were normalized to one million reads for each sample for comparison, and then aggregated and averaged on the repeat elements to show the global difference between conditions. For ChIP-Seq signals around Refseq genes,

we defined a distance scaled signal, *p*_*i*_, to represent the ChIP-seq signal intensity,

$$p_{i}=\sum_{k}w_{k}s_{k}$$

*p*_*i*_ is the weighted sum of ChIP-seq reads *s*_*k*_ at genomic positions *k*, where their weights decrease with distance from the TSS of transcription *i*.

In this definition,

$$w_{k}=\frac{2e^{-µ\left|k-t_{i}\right|}}{1+e^{-µ\left|k-t_{i}\right|}}$$

and *t*_*i*_ is the genomic position of the TSS for transcript *i*. The parameter *μ* determine the decay rate, which is a function of distance from the TSS. For H3K4me3 and H3K27me3 markers, we set *μ* to by 2 kb from the TSS, and then the contribution of corresponding signal has decayed to half of that at the TSS.

### ChIP-Seq clustering analysis

We use K-means clustering to classify distance scaled H3K9me3 ChIP-Seq signal on Refseq genes between four conditions: CC, NT two-cell, Kdm4b-injected NT two-cell and WT two-cell embryos, setting k=7. A group with gene number less than 100 and with extremely high level of H3K9me3 signal was excluded from the analysis. FPKM of the genes in each cluster were plotted to check the expression pattern, only genes with averaged FPKM ⩾1 were counted in the analysis and performed functional enrichment. Significance of Kdm4b de-repressed genes enrichment in each cluster was calculated using Hypergeometric test with expressed genes as background.

### Gene ontology analysis

Functional annotation was performed using the Database for Annotation, Visualization and Integrated Discovery (DAVID) Bioinformatics Resource [41]. Gene ontology terms for each function cluster were summaries to a representative term and *P*-values were plotted to show the significance.

### Accession numbers

The Gene Expression Omnibus (GEO) accession numbers for the single-cell RNA-seq, ULI-NChIP-seq datasets are GSE70605, GSE70606, and GEO number for the single-cell RRBS is GSE70607.
