# Supplementary material for: Identification of key factors conquering developmental arrest of somatic cell cloned embryos by combining embryo biopsy and single-cell sequencing
Source: Cell Discov. 2016 Jun 7;2:16010–. doi: 10.1038/celldisc.2016.10 (PMC4897595; doi:10.1038/celldisc.2016.10)
Supplement: Supplementary Figure S1 [file celldisc201610-s1.pdf]

Supplementary Figure S1 Quality control of single-cell RNA-Seq data.

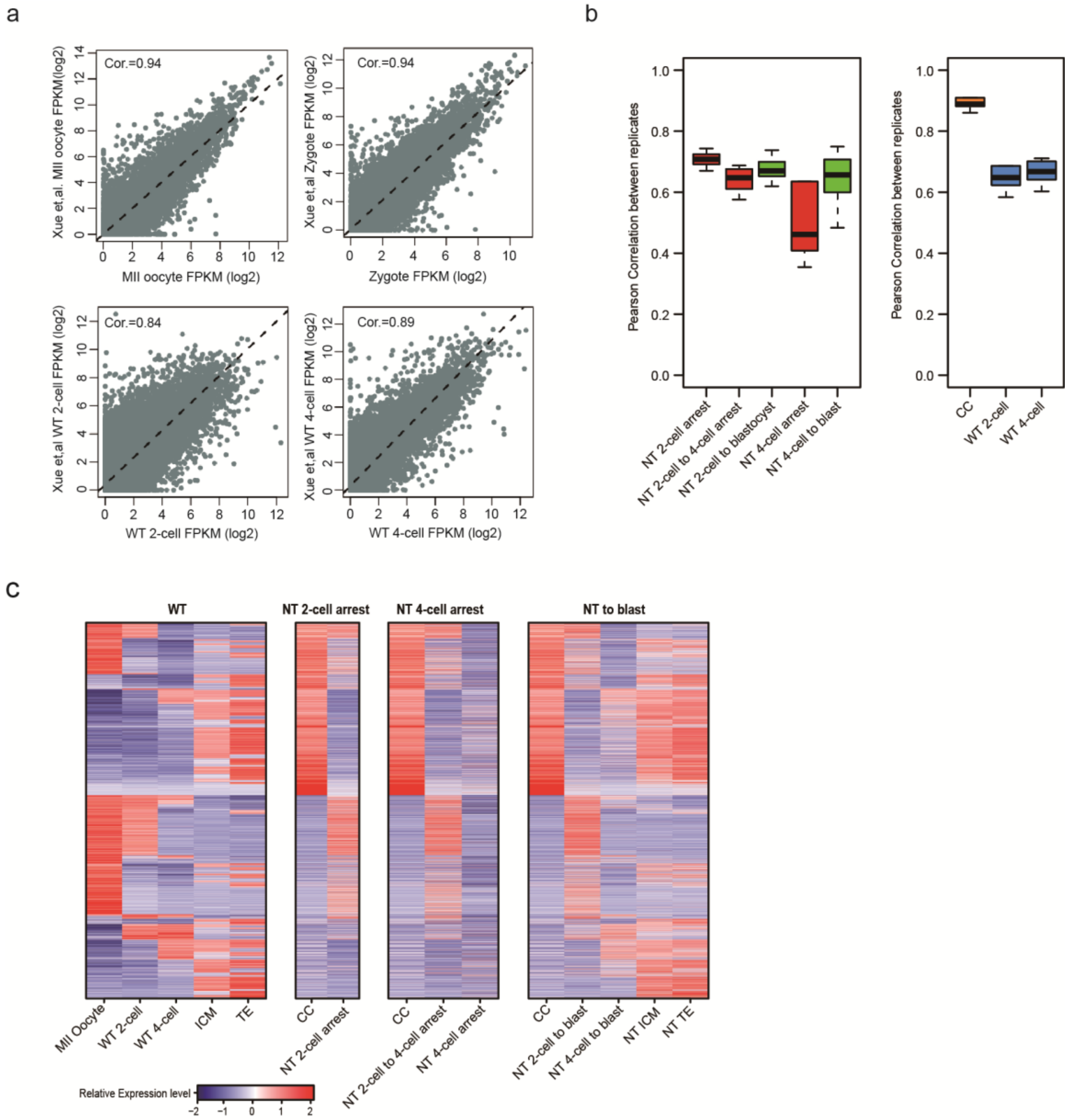

(a) Pearson correlation with RNA-Seq datasets at each indicated developmental stage. Correlations were calculated based on FPKM of all Refseq transcripts. Our samples were highly consistent with the published dataset.

(b) Boxplot showing the Pearson correlations between each individual single-cell transcriptome within the sample types. The single-cell transcriptome were highly reproducible, except for NT 4-cell arrest samples, which might have abnormal transcriptome profiles in great diversity.

(c) Genome-wide expression roadmap of WT and SCNT embryos. Heatmap showed relative gene expression levels during embryogenesis of WT and SCNT embryos with different developmental fates. Genes were clustered using k-means clustering, and only genes with averaged FPKM≥1 were used in this analysis.
