# Supplementary material for: Identification of key factors conquering developmental arrest of somatic cell cloned embryos by combining embryo biopsy and single-cell sequencing
Source: Cell Discov. 2016 Jun 7;2:16010–. doi: 10.1038/celldisc.2016.10 (PMC4897595; doi:10.1038/celldisc.2016.10)
Supplement: Supplementary Figure S2 [file celldisc201610-s2.pdf]

Supplementary Figure S2 PCA analysis on SCNT and WT embryos at 2-cells or 4-cells stage.

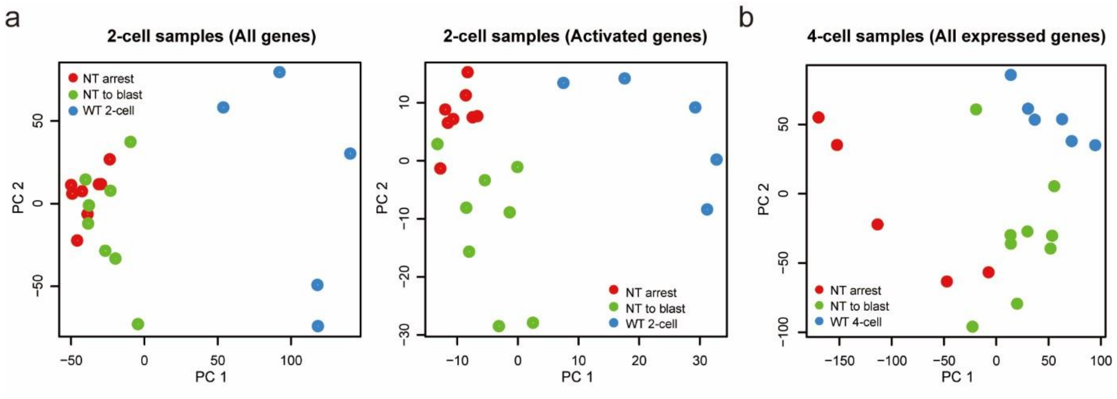

(a) PCA analysis of 2-cell samples for all expressed genes (left panel) and ZGA related genes (right panel). Genes with average FPKM ≥ 1 across MII and all 2-cell samples were defined as all expressed genes. ZGA related genes were defined as up-regulated genes compared to MII Oocytes in each type of 2-cell stage samples, and the ZGA genes in each condition were pooled to form the final ZGA gene list (3736 genes). Colors represent different types of samples.

(b) PCA analysis of the NT 4-cell arrest, NT 4-cell to blastocyst and WT 4-cell samples. All expressed genes were defined by averaged FPKM ≥1 across all samples of 4-cell stage. Colors represent different types of samples.
