# Supplementary material for: Identification of key factors conquering developmental arrest of somatic cell cloned embryos by combining embryo biopsy and single-cell sequencing
Source: Cell Discov. 2016 Jun 7;2:16010–. doi: 10.1038/celldisc.2016.10 (PMC4897595; doi:10.1038/celldisc.2016.10)
Supplement: Supplementary Figure S3 [file celldisc201610-s3.pdf]

Supplementary Figure S3 Kdm4b and Kdm4d expression in WT samples

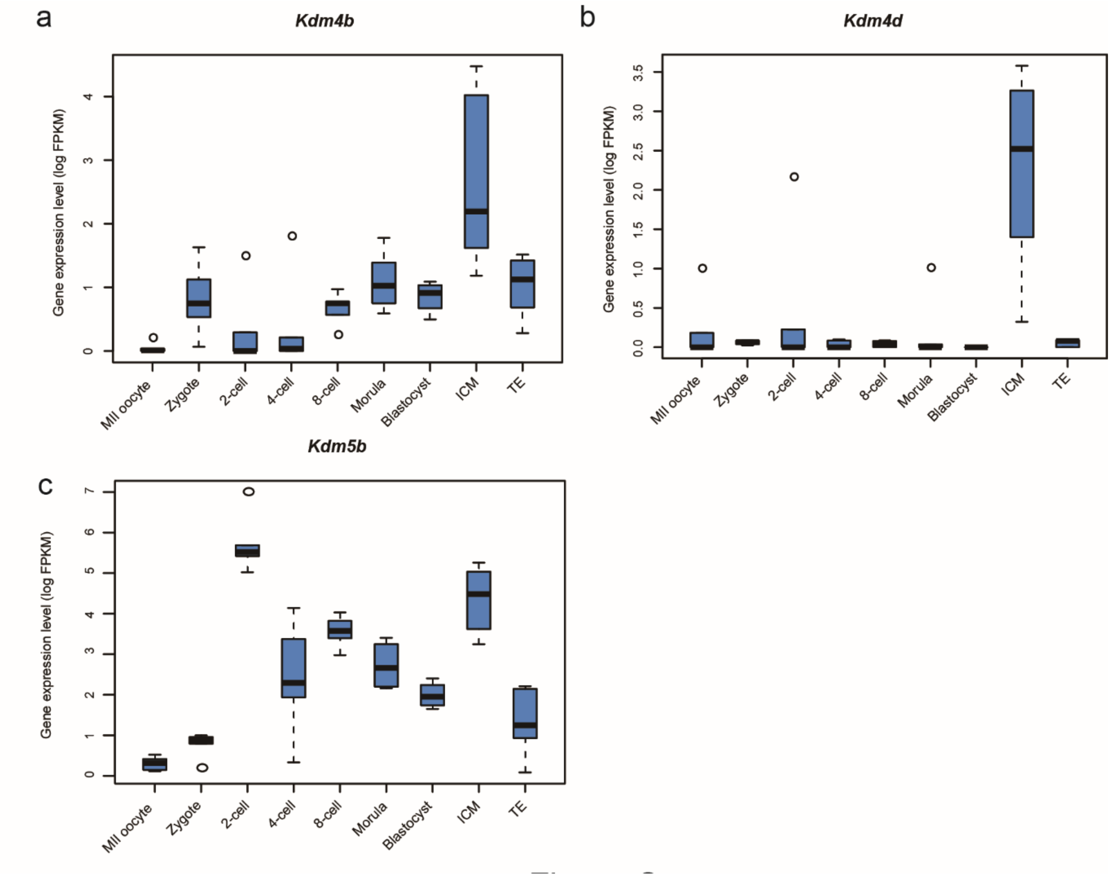

Boxplot showing the expression level of *Kdm4b* (**a**), *Kdm4d* (**b**) and *Kdm5b* (**c**) in each developmental stages of WT embryo. Expression levels were quantified to FPKM with log scale.
