# Supplementary material for: Identification of key factors conquering developmental arrest of somatic cell cloned embryos by combining embryo biopsy and single-cell sequencing
Source: Cell Discov. 2016 Jun 7;2:16010–. doi: 10.1038/celldisc.2016.10 (PMC4897595; doi:10.1038/celldisc.2016.10)
Supplement: Supplementary Figure S4 [file celldisc201610-s4.pdf]

**Supplementary Figure S4 Effective Knockdown and rescue of Kdm4b and Kdm5b in NT embryos**

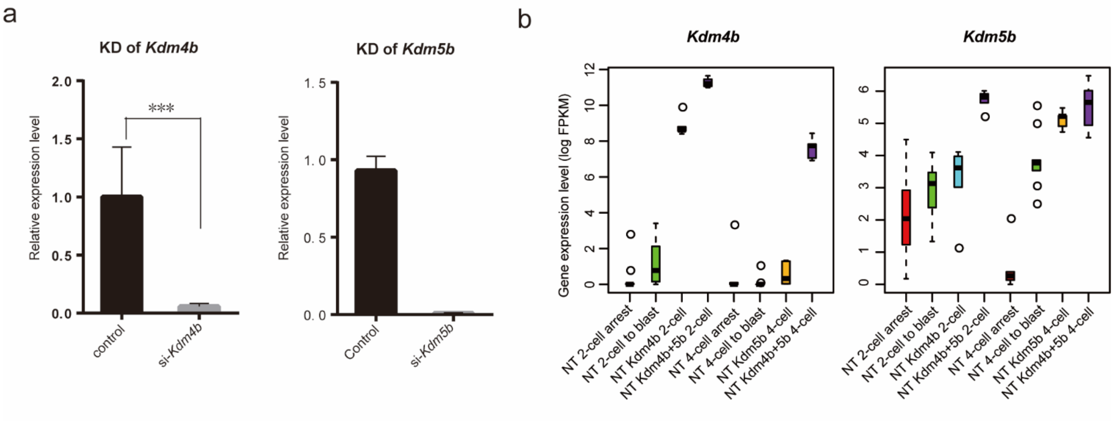


**(a)** *Kdm4b/5b* expression levels were reduced by *Kdm4b/5b* siRNA injection into embryos. RT-qPCR analysis of *Kdm4b/5b* was performed 48 h after injection. The relative expression levels of *Kdm4b/5b* relative to *H2A.Z* were compared with control embryos.

**(b)** Boxplot of *Kdm4b* and *Kdm5b* expression level in single mRNA injected or co-injection samples in 2-cell and 4-cell stage. Higher expression level indicated the successful re-introduction these mRNAs in SCNT embryos.
