# Supplementary material for: Identification of key factors conquering developmental arrest of somatic cell cloned embryos by combining embryo biopsy and single-cell sequencing
Source: Cell Discov. 2016 Jun 7;2:16010–. doi: 10.1038/celldisc.2016.10 (PMC4897595; doi:10.1038/celldisc.2016.10)
Supplement: Supplementary Figure S5 [file celldisc201610-s5.pdf]

Supplementary Figure S5 Injection of Kdm4b mRNA reduce the H3K9me3 level and restore the expression level of SCNT embryos

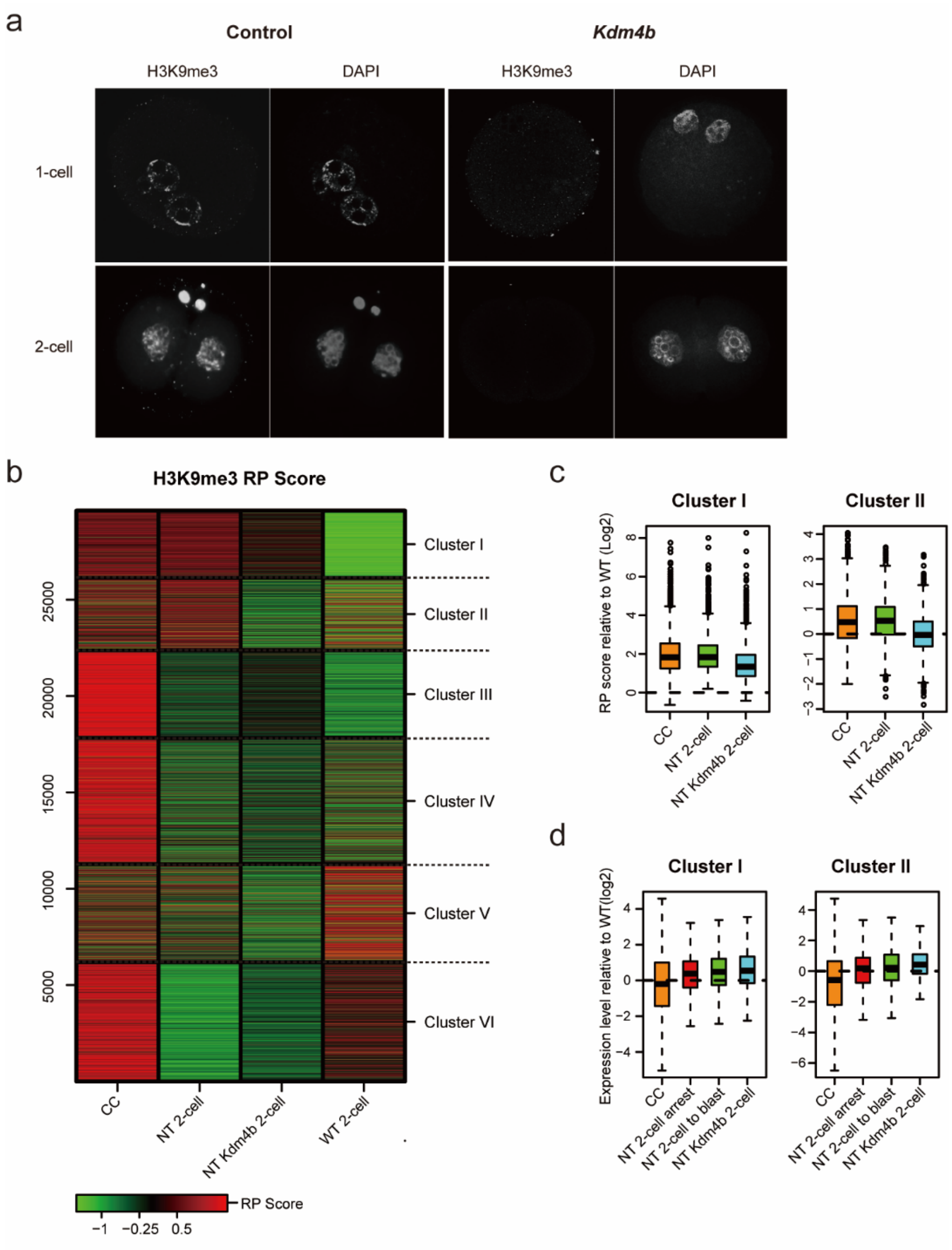

(a) Immunofluorescence analysis reveals the removal of H3K9me3 in Kdm4b mRNA-injected SCNT embryos. Both 1-cell (top) and 2-cell-stage (bottom) SCNT embryos were stained for H3K9me3, and control (left) and Kdm4b-injected (right) SCNT embryos were compared.

(b) K-means clustering of distance scaled H3K9me3 signal at promoters of all Refseq transcripts. Rows represent a promoter of Refseq transcript, and columns represent samples. Replicate of each condition were merged in this analysis. Cluster I transcripts (3481 transcripts) and Cluster II transcripts (3767) were identified as potential *Kdm4b* targets.

(c) Boxplot of normalized H3K9me3 signals of genes in Cluster I (left panel) and

Cluster II (right panel) relative to WT 2-cell samples in Supplementary Fig. S5b. The H3K9me3 signal showed a clear decrease after *Kdm4b* injected.

(d) Boxplot showing gene expression levels relative to WT 2-cell samples of Cluster I and Cluster II transcripts in Supplementary Figure S5b. Unexpressed genes (genes with averaged FPKM$\leqslant$1) were removed from this analysis. Expression level of the SCNT samples was subtracted by the expression level of WT 2-cell samples for visualization.
