# Supplementary material for: Identification of key factors conquering developmental arrest of somatic cell cloned embryos by combining embryo biopsy and single-cell sequencing
Source: Cell Discov. 2016 Jun 7;2:16010–. doi: 10.1038/celldisc.2016.10 (PMC4897595; doi:10.1038/celldisc.2016.10)
Supplement: Supplementary Figure S6 [file celldisc201610-s6.pdf]

Supplementary Figure S6 ESCs and mouse derived from rescued NT embryos

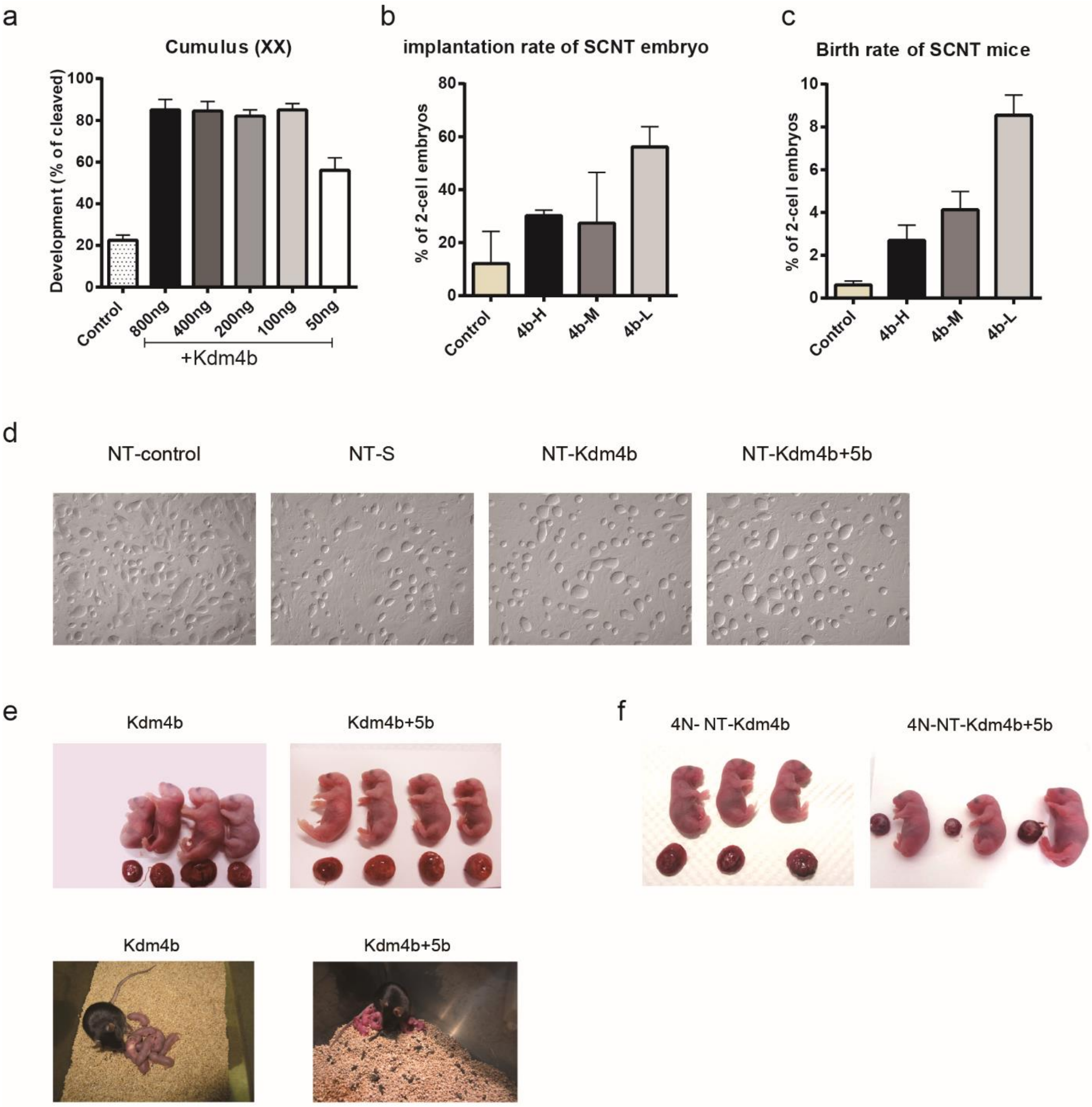

(a) Preimplantation developmental rate of SCNT embryos injected with diluted *Kdm4b* mRNA.

(b-c) Injection of a lower concentration of *Kdm4b* mRNA greatly improved the implantation (**b**) and birth (**c**) rates of SCNT embryos. High, middle and low concentrations of *Kdm4b* mRNA were used (800, 200, and 100 ng/µl, respectively). The concentration of *Kdm4b* in following experiment is 100ng/µl.

(d) Representative images of ntES cell lines produced from the cloned blastocysts.

(e-f) Fetuses produced from injected SCNT (**e**) or NT-tetraploid-aggregated embryos (**f**). The cloned mice from injected SCNT (**e**) could grow to adulthood with normal fecundity.
