# Supplementary material for: Identification of key factors conquering developmental arrest of somatic cell cloned embryos by combining embryo biopsy and single-cell sequencing
Source: Cell Discov. 2016 Jun 7;2:16010–. doi: 10.1038/celldisc.2016.10 (PMC4897595; doi:10.1038/celldisc.2016.10)
Supplement: Supplementary Figure S7 [file celldisc201610-s7.pdf]

Supplementary Figure S7 Quality control of single cell RRBS-Seq

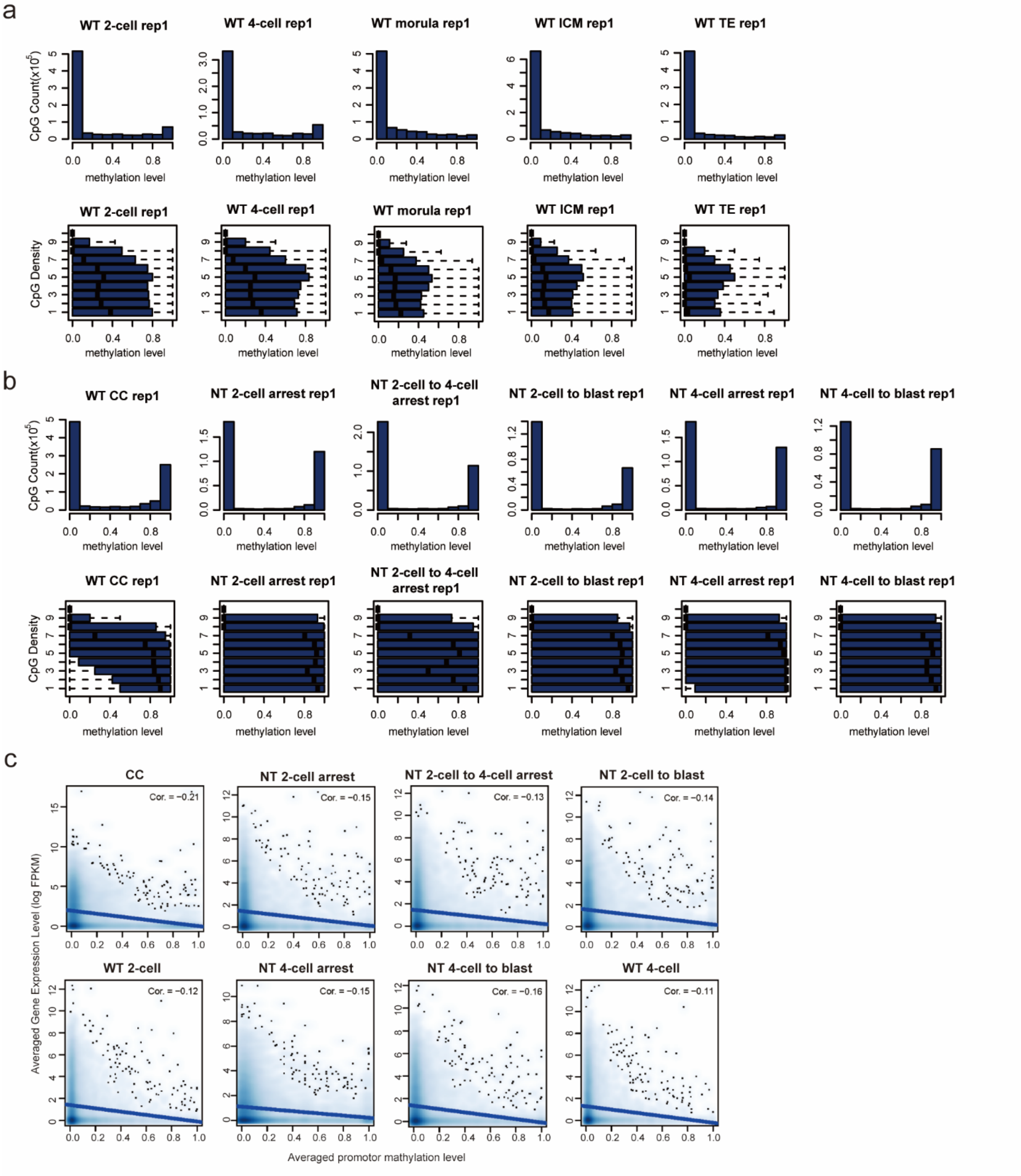

(a) CpG methylation level distribution for WT lineages and NT samples. Representative samples are shown for each type of embryo. CpG sites were binned based on the methylation levels for each sample. The *y*-axis shows the number of CpG sites in each methylation bin.

(b) Methylation levels across local CpG densities for WT lineages and NT samples. CpG sites were binned based on local CpG density within 100-bp tiles; dark blue highlights the average methylation levels of each bin.

(c) Correlation of promoter methylation levels and gene expression levels in 2-cell (upper panel) and 4-cell (lower panel) samples. The *x*-axis represents the average methylation levels of RefSeq gene promoters, based on the covered CpG sites within ±2 kb around the TSS site. The *y*-axis represents the average FPKM of the

corresponding genes; a linear regression fit curve was plotted. Pearson's correlation values for methylation and expression levels were calculated and presented.
