# Supplementary material for: Identification of key factors conquering developmental arrest of somatic cell cloned embryos by combining embryo biopsy and single-cell sequencing
Source: Cell Discov. 2016 Jun 7;2:16010–. doi: 10.1038/celldisc.2016.10 (PMC4897595; doi:10.1038/celldisc.2016.10)
Supplement: Supplementary Figure S8 [file celldisc201610-s8.pdf]

Supplementary Figure S8 Correlation of DNA methylation, H3K9me3 level and gene expression level

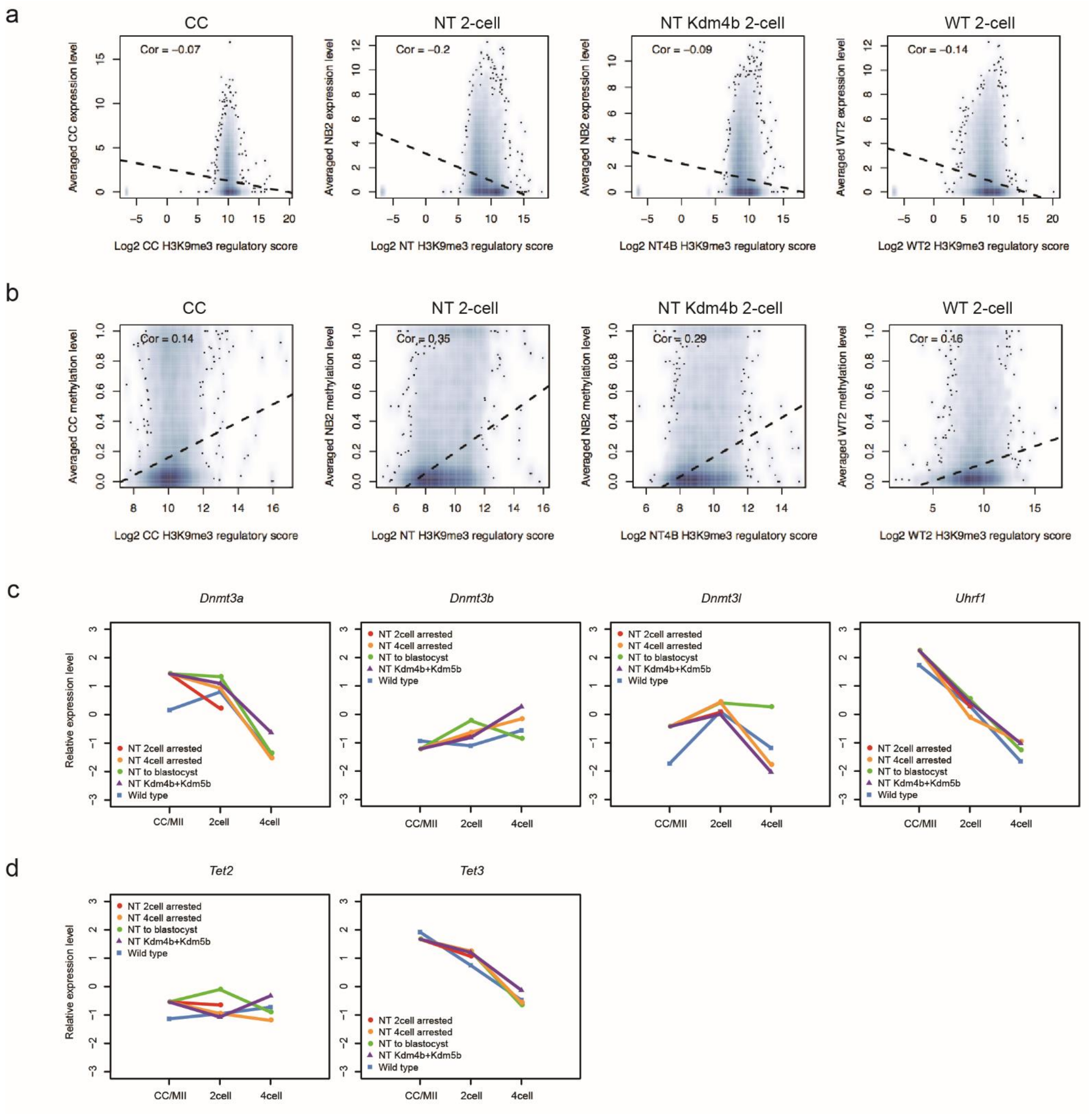

(a) Correlation of promoter H3K9me3 levels and gene expression levels in CC, NT, NT+Kdm4b and WT samples. The *x*-axis represents the distance scaled H3K9me3 levels of RefSeq gene promoters, and the *y*-axis represents the average FPKM of the corresponding genes; a linear regression fit curve was plotted. Pearson's correlation values for H3K9me3 levels and expression levels were calculated and presented.

(b) Correlation of promoter H3K9me3 levels and methylation levels in CC, NT, NT+Kdm4b and WT samples. The *x*-axis represents the distance scaled H3K9me3 levels of RefSeq gene promoters, and the *y*-axis represents the average methylation levels of corresponding promoters, based on the covered CpG sites within ±2 kb around the TSS site; a linear regression fit curve was plotted. Pearson's correlation values for methylation and expression levels were calculated and presented.

(c-d) Relative expression level of DNA methylation writers (**c**) and erasers (**d**) in SCNT

and WT samples. Expression levels were quantified using FPKM and then normalized based on z-score of different sample types. Transcript with overall highest FPKM was used as the FPKM of the genes.
