# Supplementary material for: Identification of key factors conquering developmental arrest of somatic cell cloned embryos by combining embryo biopsy and single-cell sequencing
Source: Cell Discov. 2016 Jun 7;2:16010–. doi: 10.1038/celldisc.2016.10 (PMC4897595; doi:10.1038/celldisc.2016.10)
Supplement: Supplementary Table S1 [file celldisc201610-s9.pdf]

Supplementary Table S1

Gene lists of the blastocyst-high genes which may account for 2-cell arrest of cloned embryos.

NM_175300	Anapc2	NM_001081049	Kmt2a	NM_199042	Thap1
NM_008554	Ascl2	NM_001033276	Kmt2d	NM_001081102	Whsc1
NM_138679	Ash1l	NM_013594	Mbd1	NM_011728	Xpa
NM_030178	Brpf1	NM_010774	Mbd4	NM_009541	Zbtb17
NM_178576	Cpsf4	NM_013729	Mixl1	NM_001276332	Zbtb34
NM_007837	Ddit3	NM_023290	Mkrn2	NM_198248	Zbtb40
NM_178683	Depdc1b	NM_016662	Mxd3	NM_029334	Zc3h14
NM_001037937	Deptor	NM_017373	Nfil3	NM_001081016	Zc3h7b
NM_013874	Dpf1	NM_021315	Noc3l	NM_001045486	Zfp180
NM_178609	E2f7	NM_010444	Nr4a1	NM_013922	Zfp354c
NM_007920	Elf1	NM_011023	Otx1	NM_172740	Zfp420
NM_007922	Elk1	NM_013633	Pou5f1	NM_001033249	Zfp583
NM_007970	Ezh1	NM_010127	Pou6f1	NM_001033159	Zfp597
NM_001146689	Ezh2	NM_027504	Prdm16	NM_172749	Zfp646
NM_007971	Ezh2	NM_001081355	Prdm2	NM_001083958	Zfp655
NM_001291067	Foxa2	NM_144809	Prdm9	NM_146259	Zfp668
NM_010278	Gfi1	NM_016703	Preb	NM_145916	Zfp7
NM_026816	Gtf2f2	NM_028410	Prkrir	NM_175466	Zfp770
NM_008228	Hdac1	NM_001163641	Setdb1	NM_001037745	Zfp791
NM_010437	Hivep2	NM_001163642	Setdb1	NM_145612	Zfp810
NM_010495	Id1	NM_018877	Setdb1	NM_001200023	Zfp963
NM_016849	Irf3	NM_001081024	Setdb2	NM_009577	Zik1
NM_018826	Irx5	NM_013672	Sp1	NM_001081329	Zkscan2
NM_178637	Kat5	NM_001013817	Sp140	NM_001013765	Zscan4c
NM_026370	Kat8	NM_001163018	Suz12	NM_001100186	Zscan4d
NM_172132	Kdm4b	NM_199196	Suz12		
NM_013692	Klf10	NM_031182	Tfap4		
