# Supplementary material for: Identification of key factors conquering developmental arrest of somatic cell cloned embryos by combining embryo biopsy and single-cell sequencing
Source: Cell Discov. 2016 Jun 7;2:16010–. doi: 10.1038/celldisc.2016.10 (PMC4897595; doi:10.1038/celldisc.2016.10)
Supplement: Supplementary Table S2 [file celldisc201610-s10.pdf]

Supplementary Table S2

Sequence for siRNA and PCR

**Target sequences of siRNA**			
Gene	Name	Target Sequence(5'→3')	Reference
Kdm4b	Kdm4b-si-1	GCCCATGTGTACTTGGAAATT	[1]
	Kdm4b-si-1	GCCCATTATCCCAATGCTGTA	
Kdm5b	Kdm5b-si-1	CTCCGATACATGATTGAAAtt	[2]
	Kdm5b-si-2	TCTTTGCCCTCGGTGTGAC	[3]
	Kdm5b-si-3	GAGATGCACTCCGATACAT	
**Realtime PCR primers**			
mRNA	Strand	Sequence (5'→3')	Reference
Kdm4b	F	CAATGCTGTACGTGTTGCCC	
	R	AAGGGGTTGTTCCCTGTGAG	
Kdm5b	F	AACCGTGAACTGGCAACATA	
	R	AGTCCAGAACCCACTTGGTC	
Gapdh	F	CATGGCCTTCCGTGTTCCTA	
	R	GCCTGCTTCACCACCTTCTT	
**cDNA-cloning primers**			
T7-flag	F	TAATACGACTCACTATAGGGATGGACTACAAAGACGATGACGATAAA	
Kdm4a	F	CTACAAAGACGATGACGATAAAATGGCTTCTGAATCAGAAACTTTGAAC	
	R	CTACTCCATGATGGCCCGGTA	
Kdm4b	F	ATGGACTACAAAGACGATGACGATAAAGGGTCCGAGGACCACAGC	
	R	CTAGAAGGGTGCTCCAGGCC	
Kdm4d	T7-F	TAATACGACTCACTATAGGATGAAGACGAAGTCCACATGTGCTC	
	R	TTAGGGGTCGGAGGTCAATCCC	
Kdm5b	T7-F	TAATACGACTCACTATAGGATGAAGACGAAGTCCACATGTGCTC	
	R	TTACTTTCGGCTTGGTGCGTC	
GFP	T7-F	TAATACGACTCACTATAGGGATGGTGAGCAAGGGCGAGG	
	R	TTACTTGTACAGCTCGTCCATGCCGAG	

[1] DAS P P, SHAO Z, BEYAZ S, et al. Distinct and combinatorial functions of Jmjd2b/Kdm4b and Jmjd2c/Kdm4c in mouse embryonic stem cell identity [J]. Molecular cell, 2014, 53(1): 32-48.
[2] MA P, PAN H, MONTGOMERY R L, et al. Compensatory functions of histone deacetylase 1 (HDAC1) and HDAC2 regulate transcription and apoptosis during mouse oocyte development [J]. Proceedings of the National Academy of Sciences of the United States of America, 2012, 109(8): E481-9.
[3] XIE L, PELZ C, WANG W, et al. KDM5B regulates embryonic stem cell self-renewal and represses cryptic intragenic transcription [J]. The EMBO journal, 2011, 30(8): 1473-84.
